# Transition-state stabilization in *Escherichia coli* ribonuclease P RNA-mediated cleavage of model substrates

**DOI:** 10.1093/nar/gkt853

**Published:** 2013-10-03

**Authors:** Shiying Wu, Yu Chen, Guanzhong Mao, Stefan Trobro, Marek Kwiatkowski, Leif A. Kirsebom

**Affiliations:** ^1^Department of Cell and Molecular Biology, Box 596, Uppsala University, SE-751 24 Uppsala, Sweden, ^2^Department of Chemistry, University of Michigan, Ann Arbor, MI 48109, USA and ^3^Department of Molecular Biology, Swedish University of Agricultural Sciences, Box 590, SE-751 24 Uppsala, Sweden

## Abstract

We have used model substrates carrying modified nucleotides at the site immediately 5′ of the canonical RNase P cleavage site, the −1 position, to study *Escherichia coli* RNase P RNA-mediated cleavage. We show that the nucleobase at −1 is not essential but its presence and identity contribute to efficiency, fidelity of cleavage and stabilization of the transition state. When U or C is present at −1, the carbonyl oxygen at C2 on the nucleobase contributes to transition-state stabilization, and thus acts as a positive determinant. For substrates with purines at −1, an exocyclic amine at C2 on the nucleobase promotes cleavage at an alternative site and it has a negative impact on cleavage at the canonical site. We also provide new insights into the interaction between *E. coli* RNase P RNA and the −1 residue in the substrate. Our findings will be discussed using a model where bacterial RNase P cleavage proceeds through a conformational-assisted mechanism that positions the metal(II)-activated H_2_O for an in-line attack on the phosphorous atom that leads to breakage of the phosphodiester bond.

## INTRODUCTION

**Figure 1.**
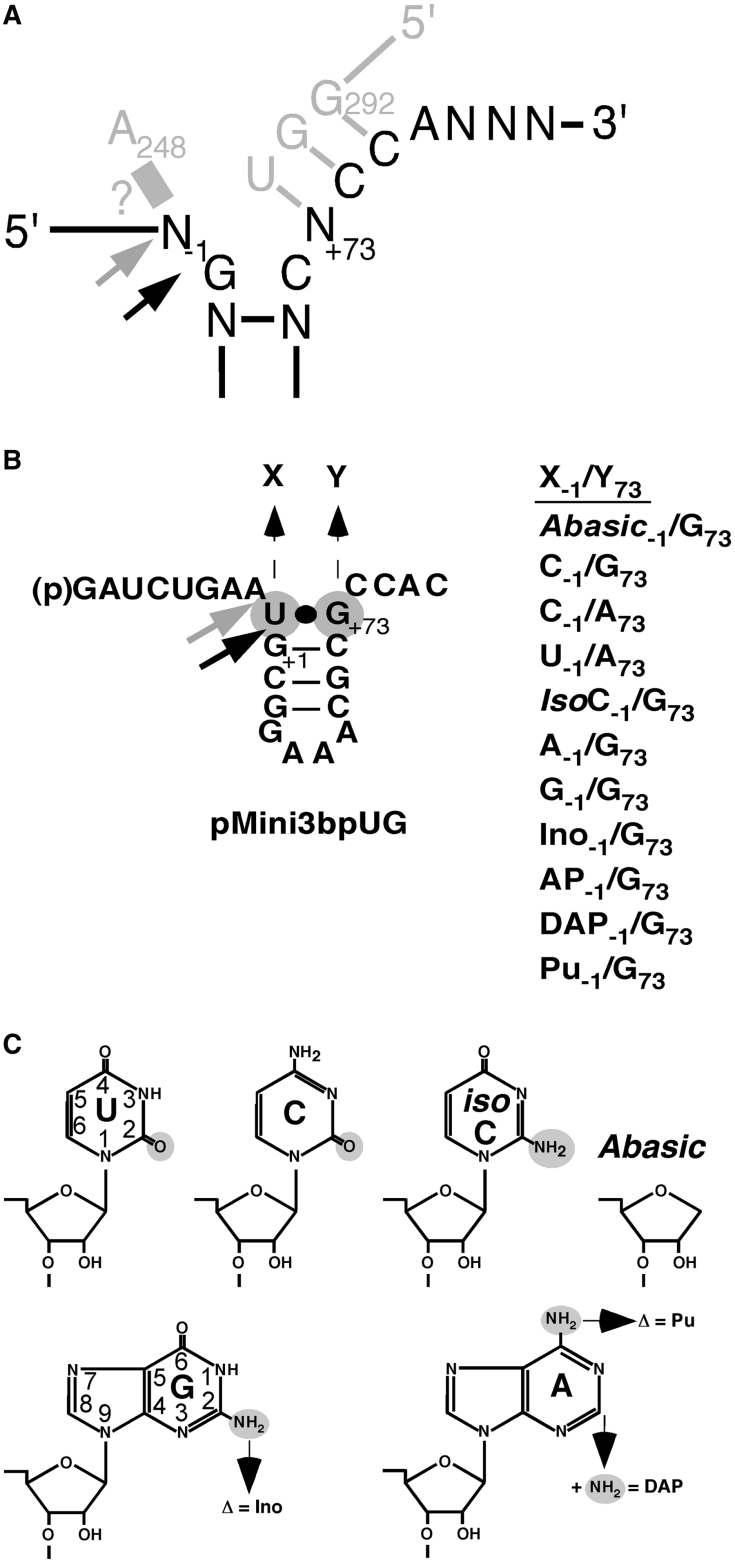
Structures of the model hairpin loop substrate pMini3bp and structure of uridine, cytidine, *iso*-cytidine, guanosine and adenosine. (**A**) Illustration of the N_−1_/_248 _- and the RCCA-RPR interactions [interacting residues underlined (6,9,10)]. Substrate residues are marked in black and residues marked in grey represent the RPR. Black and grey arrows mark the canonical and the alternative cleavage sites, respectively. ? indicate if and how residues −1 and 248 interact (see the main text). (**B**) Structure of pMini3bpUG and its derivatives. The black arrow marks the canonical cleavage site at +1 and the grey arrow marks the alternative cleavage site −1. X and Y indicate where base changes were introduced as indicated on the right side. *Abasic*, deletion of the base; *Iso*C, *iso*-cytidine; Ino, inosine; 2AP, 2-amino purine; DAP, 2;6-diamino purine; Pu, purine. (**C**) Structure of the bases uridine (U), cytidine (C), iso-cytidine (*Iso*C), *Abasic*, guanosine (G) and adenosine (A). Chemical groups marked in small grey circles refer to the groups that were substituted or deleted as indicated. The ring numbering for U and G is as indicated. For details see text.

Transfer RNA (tRNA) genes are transcribed as precursors with extra residues at their 5′- and 3′-ends that have to be removed to generate functional tRNAs. The endoribonuclease—RNase P—is responsible for removing the extra 5′ residues, i.e. the 5′ leader. In Bacteria, RNase P consists of one protein and one RNA subunit, referred to as the C5 protein and RNase P RNA (RPR), respectively; in Archaea and Eukarya, the number of proteins is expanded. Irrespective of origin, the catalytic activity resides in the RNA moiety and the RNA alone can mediate cleavage in the absence of the protein (1–3). However, recent data suggest the existence of RNase P-like activities based solely on protein (4,5). Several determinants in the tRNA precursor substrate influence binding and cleavage efficiency [for recent reviews see (6–8)]. For one of these, the residue at −1, genetic and biochemical data suggest that it interacts with a conserved A at position 248 in *Escherichia coli* (*Eco*) RPR_wt_. This interaction is referred to as the N_−__1_/A_248_ interaction [(6,9); see Figure 1A]. The recently published structure of RNase P in complex with tRNA positions A_248_ close to the 5′ termini of the tRNA (11); however, it is still unclear if and how N_−__1_ in the substrate interacts with A_248_.

The identity of the −1 residue varies in tRNA precursors. In *E. coli*, the majority (≈57%) carry a uridine at −1, whereas in others, e.g. *Mycobacterium tuberculosis*, a cytidine is more frequent (12,13). In a tRNA precursor context, *in vitro* data suggest that substrates with U at −1 bind to RPR with higher affinity compared with substrates with other −1 residues (9,14). Moreover, for substrates with C at −1, which can pair with a guanosine at position +73 [the discriminator base position; (15)], *in vitro* and *in vivo* studies show that cleavage occurs at the correct site between positions −1 and +1 (the canonical site or the +1 site), as well as at an alternative site between residues at −1 and −2, i.e. at −1 (6–8,16). Cleavage of substrates with C_−__1_/G_+73_ and U_−1_/G_+73_ pairs has furthermore demonstrated that optimal cleavage of the former substrate requires higher Mg^2+^-concentrations (14). The rate of cleavage for *Eco* RPR_wt_ as a function of the identity of the residue at −1, however, does not change to any significant extent in a tRNA precursor context (9,14). In contrast, for model hairpin loop substrates (see Figure 1B), the −1 identity influences the rate of cleavage (6,7,13,14,17). Hence, the effect of the −1 residue in a pre-tRNA context is likely to be obscured by other elements affecting RPR-mediated cleavage (14). Moreover, there is no information about the contribution of the chemical groups on the −1 base to catalysis. Therefore, we decided to reinvestigate the impact of the identity of the −1 residue on cleavage using a model substrate. Our specific objectives were to identify chemical groups of the −1 nucleobase that contribute to catalysis and to determine if the nucleobase at −1 is absolutely necessary for cleavage. As available data suggest that the protein subunit of RNase P does not interact with the residue at −1 (6–8), we studied cleavage mostly in the absence of the protein. Our results show that the nucleobase at the −1 position is not necessary for cleavage in the context of a model hairpin loop substrate. However, the presence, identity and specific chemical groups at −1 influence cleavage site selection and the kinetics of cleavage. The −1 position contributes to the stabilization of the transition state for cleavage at the canonical cleavage site. We will discuss our data in view of the recently solved structure of RNase P in complex with tRNA (11) and a model that emphasizes the importance of the positioning of Mg(II) at and near the cleavage site for ensuring cleavage at the correct site.

## MATERIALS AND METHODS

### Preparation of substrates, RPRs and C5 protein

The different pMini3bp derivatives except pMini3bp*Abasic*G and pMini3bp*Iso*CG were purchased from Dharmacon (Lafayette, CO, USA). The pMini3bp*Abasic*G and pMini3bp*Iso*CG were synthesized in-house essentially according to Wincott *et al.* (18). The rSpacer and isoC phosphoramidites were purchased from MedProbe AS, Norway (Glen Research, USA) and ChemGenes, USA, respectively. All the RNA substrates were purified on 15% denaturing polyacrylamide gels and extracted overnight using a Biotrap device following the manufacturer’s procedures (Schleicher and Schuell, GmbH, Germany; Elutrap in USA and Canada). This was followed by phenol-chloroform extraction according to standard procedures. The different substrates were labeled with ^32^P at the 5′-end with [γ-^32^P]ATP as described elsewhere (14).

The construction of the *Eco* RPR_G248_ encoding gene has been reported elsewhere (19). The *Eco* RPR_wt_ and *Eco* RPR_G248_ were generated as T7 RNA polymerase run-off transcripts (20), whereas the C5 protein was purified from an *E. coli* BL21(DE3) strain harbouring the plasmid pET33b carrying the His6-C5 gene (N-terminal fusion). Briefly, the pET33b plasmid with the His6-C5 gene behind an isopropyl β-D-1-thiogalactopyranoside-inducible promoter was transformed into *E. coli* BL21(DE3). The cells were grown at 37°C in LB liquid medium supplemented with 50 µg/ml kanamycin. At OD_600_ = 0.6, isopropyl β-D-1-thiogalactopyranoside was added to a final concentration of 1.5 mM and the culture was incubated for another 4 h. The His6-C5 protein was purified essentially as described by Feltens *et al.* (21), and the protein concentration was determined using the standard Bradford protein assay.

### Assay conditions—cleavage by RPR alone and in the presence of the C5 protein

The RPR alone reactions were conducted in buffer C [50 mM Mes (pH 6.1 at 37°C), 0.8 M NH_4_Cl] and indicated Mg(OAc)_2_ concentrations. Before adding the preheated (37°C) substrate, the RPRs were pre-incubated at 37°C in buffer C and Mg(OAc)_2_ for at least 10 min to allow proper folding. In the Mg^2+^ titration experiments, the concentrations of substrates were 0.02 µM while the concentration of RPRs varied between 0.8 and 5.2 µM [the concentration varied depending on substrates and RPR combination, see Figure legend 3; (22)].

All the reactions with the His6-C5 protein were carried out in buffer A [50 mM Tris–HCl (final pH 7.2), 5% (w/v) PEG 6000, 100 mM NH_4_Cl] supplemented with 10 mM MgCl_2_. The RPR was pre-incubated in buffer A at 37°C for 10 min. The His6-C5 was added, and incubation was continued for an additional 10 min followed by addition of preheated (37°C) substrate. The concentrations of RPR and His6-C5 were 0.004 µM and 0.21 µM [empirically determined; see also e.g. (23)], respectively, and the concentration of substrate was 0.02 µM.

To terminate the reactions, double volumes of stop solution were added (10 M urea, 100 mM EDTA).

### Determination of the kinetic constants under single-turnover conditions

The kinetic constants k_obs_ and k_obs_/K^sto^ (=k_cat_/K_m_) were determined under saturating single-turnover conditions in buffer C at pH 6.1 and 800 mM Mg(OAc)_2_. At this pH, the chemistry of cleavage is rate-limiting (24). The final concentration of the substrate was 0.02 µM, and the concentration of RPRs varied between 0.8 and 29 µM, depending on the substrate–RPR combination. Given that the substrates were labeled at the 5′-end, we used the 5′ cleavage fragments in our activity measurements. While calculating the rate, the incubation times for each substrate and RPR combination were adjusted to ensure that the velocity measurements were in the linear range, i.e. ≤40% of the substrate had been consumed.

The k_obs_ and k_obs_/K^sto^ values were obtained by linear regression from Eadie–Hofstee plots as described elsewhere [(25); see also (26,27)].

## RESULTS

**Scheme 1.**
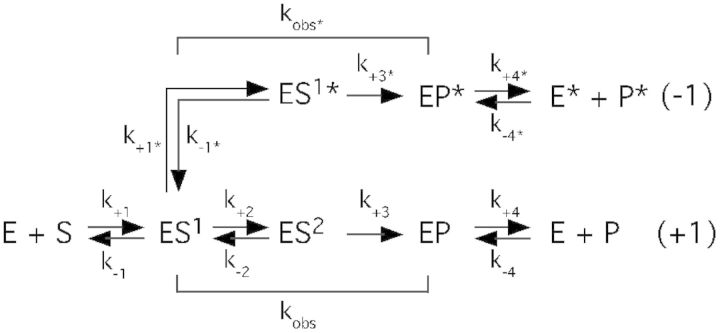

To test the contribution of residue −1 to cleavage, we used the short model substrate, pMini3bp [Figure 1; (12)], in which cleavage relies on residues near the cleavage site, mainly the N_−1_/A_248_ and the RCCA-RPR interactions (interacting residues underlined, Figure 1A; 6,9,10). For this purpose, we generated pMini3bp variants carrying changes at −1 (Figure 1B). These substrates were studied with respect to (i) cleavage site recognition, where cleavage between −1 and +1 is referred to the canonical or correct site (Figure 1) and (ii) Mg^2+^ dependence. We also determined the kinetic constants k_obs_ and k_obs_/K^sto^ (k_obs_/K^sto ^= k_cat_/K_m_; see later in the text) for cleavage under saturating-single turnover conditions at pH 6.1. At this pH, previous data have suggested that the cleavage of other model hairpin loop substrates is rate-limiting [see e.g. Ref. (24)]. In the simplified scheme the kinetic constant k_obs_ reflects the rate of cleavage as indicated while the rate constant k_obs_/K^sto ^= k_+1_ (Scheme 1). Under these conditions, we argue that K^sto ^≈ K_d_ because k_−1_ >> k_obs_ in the cleavage of pMini3bpUG by *Eco* RPR_wt_, where k_−1_ corresponds to the rate of dissociation (Supplementary Figure S1; for experimental details see Supplementary data and Refs (25,28,29); we assume that this is the case for all the pMini3bp variants). Going from ES^1^ to ES^2^ involves breaking the −1/+73 base pair in the substrate (e.g. as when C is at −1 and G at +73; see Figure 1B) and positioning of the Mg^2+^ responsible for generating the nucleophile. Furthermore, the ES^1^ to ES^2^ route results in cleavage at the canonical cleavage site +1, whereas ES^1^ to ES^1*^ describes the pathway (and e.g. does not involve breaking of the −1/+73 pair if present) that gives cleavage at the alternative site −1 [see Figure 1; see also (19)].

### The nucleobase at −1 is not essential for cleavage

**Figure 2.**
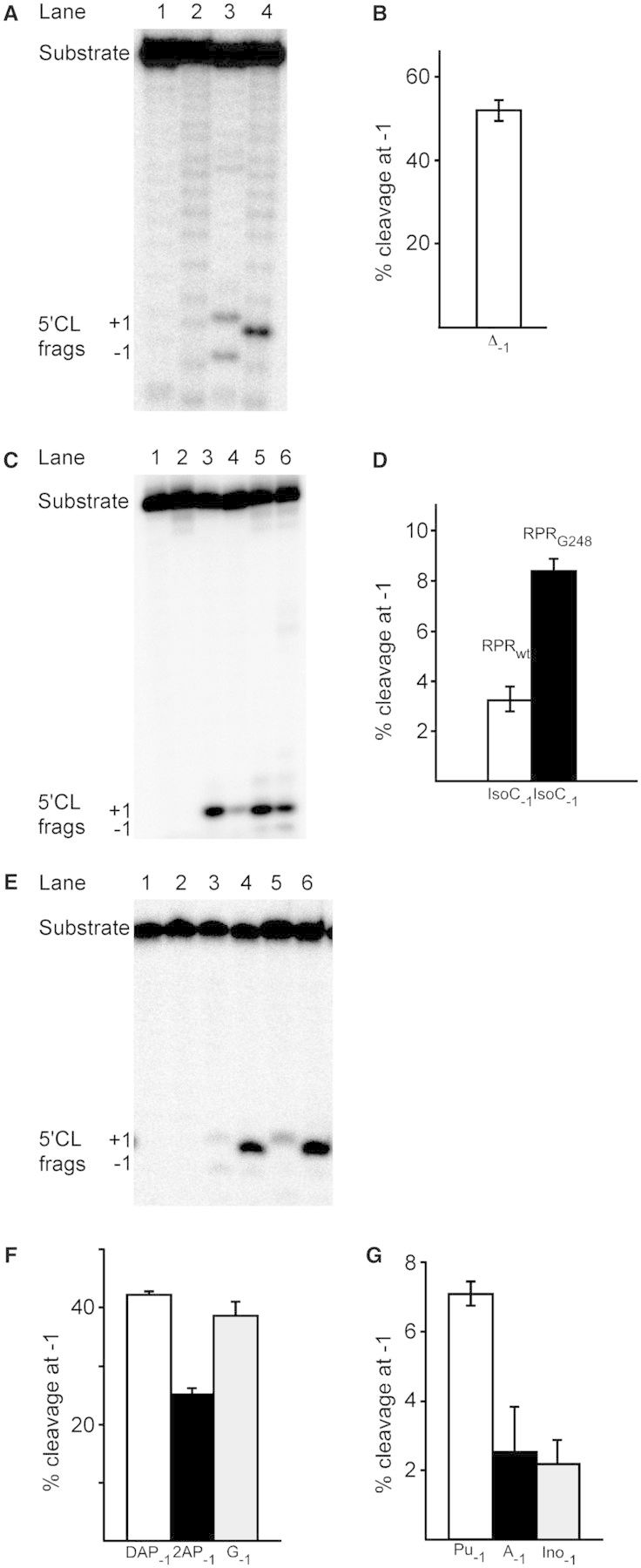
Cleavage of pMini3bp variants with *Eco* RPR. (**A**) pMini3bpUG and pMini3bp*Abasic*G; the reaction was performed at 37°C in buffer C containing 800 mM Mg^2+^ (see ‘Materials and Methods’). Lanes 1 (pMini3bp*Abasic*G) and 2 (pMini3bpUG), controls incubation without *Eco* RPR_wt_; lanes 3 (pMini3bp*Abasic*G) and 4 (pMini3bpUG) incubation with *Eco* RPR_wt_ for 1179 min and 20 s, respectively. The concentrations of *Eco* RPR_wt_ and substrate were ≈0.8 and 0.02 µM, respectively. The 5'CL frags indicate the 5′ cleavage fragments generated after cleavage at +1 and −1. The difference in migration of the +1 cleavage fragments in lanes 3 and 4 is likely because of the absence of the −1 nucleobase in the 5′ cleavage product after cleavage of pMini3bp*Abasic*G at +1. For experimental details see ‘Materials and Methods’. (**B**) Frequency of cleavage of pMini3bp*Abasic*G at the alternative site −1 with *Eco* RPR_wt_ at 800 mM Mg^2+^. For the calculations of the percentage of cleavage, we used the 5′ cleavage fragments and the data are the mean and experimental errors of at least three independent experiments. The error bars indicate experimental errors and the frequencies of cleavage at −1 were calculated as described previously (30). For experimental details, see figure legend 2A. (**C**) Cleavage of pMini3bpUG and pMini3bp*Iso*CG with *Eco* RPR_wt_ and *Eco* RPR_G248_; the reaction was performed at 37°C in buffer C containing 800 mM Mg^2+^ (see ‘Materials and Methods’). Lanes 1 (pMini3bpUG) and 2 (pMini3bp*Iso*CG), controls incubation without *Eco* RPR for 90 min; lanes 3 and 4, pMini3bpUG incubated with *Eco* RPR_wt_ and *Eco* RPR_G248_, respectively, for 20 s; lanes 5 and 6, pMini3bp*Iso*CG incubated with *Eco* RPR_wt_ (60 min) and *Eco* RPR_G248_ (90 min), respectively. The concentrations of *Eco* RPR and substrate were ≈0.8 and 0.02 µM, respectively. The 5'CL frags indicate the 5′ cleavage fragments generated after cleavage at +1 and −1. (**D**) Frequency of cleavage of pMini3bp*Iso*CG at the alternative site −1 (mean and experimental errors of at least three independent experiments) with *Eco* RPR_wt_ and *Eco* RPR_G248_ at 800 mM Mg^2+^. For experimental details and calculations, see figure legends 2B and 2C. (**E**) Cleavage of pMini3bpGG and pMini3bpAG with *Eco* RPR_wt_ in the absence and in the presence of C5. Lanes 1 (pMini3bpGG) and 2 (pMini3bpAG), controls incubation without *Eco* RPR_wt_ and C5 for 180 min; lanes 3 (pMini3bpGG) and 4 (pMini3bpAG) cleavage without C5 for 180 min; lanes 5 (pMini3bpGG) and 6 (pMini3bpAG) cleavage with C5 for 1 min. 5′ CL Frags +1 and −1 mark the migrations of the 5′ cleavage fragments. The reactions were carried out at 37°C and 800 mM Mg^2+^ and 10 mM Mg^2+^ as described in ‘Materials and Methods’. In the absence of C5, the concentration of *Eco* RPR_wt_ was 3.2 µM, whereas it was 0.002 µM in its presence. The substrate concentration was 0.02 µM. (**F**) Percentage of cleavage at −1 for substrates carrying 2;6-diaminopurine (DAP), 2-aminopurine (2AP) or guanosine (G) at −1 as indicated. The reactions were performed in the absence of C5 in buffer C at 800 mM Mg^2+^ as described earlier in text and in ‘Materials and Methods’. The calculations were done as described in figure legend 2B. (**G**) Percentage of cleavage at −1 for substrates carrying purine (Pu), adenosine (A) or inosine (Ino) at −1 as indicated. The reactions were performed in the absence of C5 in buffer C at 800 mM Mg^2+^ as described earlier in text and in ‘Materials and Methods’. The calculations were done as described in figure legend 2B.

**Figure 3.**
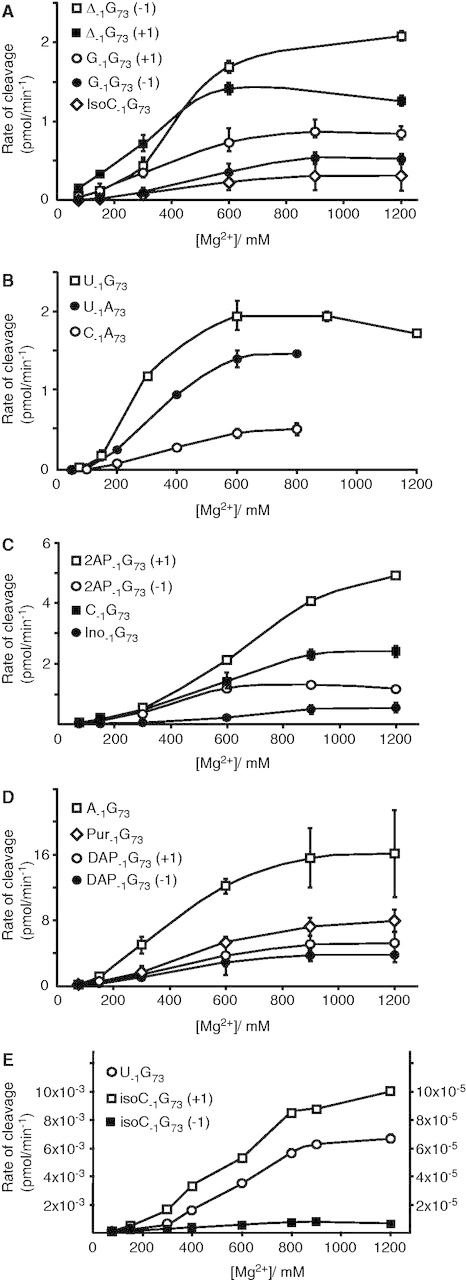
Cleavage of the pMini3bp variants by *Eco* RPR as a function of Mg^2+^ concentration. Panels A–E show the Mg^2+^ profiles for *Eco* RPR_wt_ (**A–D**) and *Eco* RPR_G248_ (**E**). The experiment was performed under single-turnover conditions at 37°C as described in ‘Materials and Methods’ and the concentration of substrates was 0.02 µM, whereas the concentration of *Eco* RPR (in parenthesis) varied according to the following: Panel A pMini3bp*Abasic*G (Δ_−1 _G_+73_, 3.2 µM); pMini3bpGG (G_−1 _G_+73_, 5.2 µM); pMini3bp*Iso*CG (IsoC_−1 _G_+73_, 3.2 µM); Panel B pMini3bpUG (U_−1 _G_+73_, 0.8 µM); pMini3bpUA (U_−1 _A_+73_, 3.1 µM); pMini3bpCA (C_−1 _A_+73_, 3.1 µM); Panel C pMini3bp2APG (2AP_−1 _G_+73_, 5.2 µM); pMini3bpCG (C_−1 _G_+73_, 1.6 µM); pMini3bpInoG (Ino_−1 _G_+73_, 5.2 µM); Panel D pMini3bpAG (A_−1 _G_+73_, 5.2 µM); pMini3bpPuG (Pur_−1 _G_+73_, 5.2 µM); pMini3bpDAPG (DAP_−1 _G_+73_, 5.2 µM); Panel E (cleavage with *Eco* RPR_G248_) pMini3bpUG (U_−1 _G_+73_, 0.8 µM; left *y*-axis); pMini3bp*Iso*CG (IsoC_−1 _G_+73_, 0.8 µM; right *y*-axis). For the calculations, we used the 5′ cleavage fragments and the data are the mean of at least three independent experiments. The bars indicate the experimental errors.

To understand if the nucleobase at −1 is necessary for cleavage, we prepared a substrate in which the base had been deleted, pMini3bp*Abasic*G (Figure 1B). *Eco* RPR_wt_ cleaved this substrate at two positions, at the canonical site +1 and at −1, with low efficiency compared with cleavage of pMini3bpUG where the base is present (Figure 2A). Cleavage of pMini3bp*Abasic*G at +1 and at −1 occurred throughout the entire concentration range of Mg^2+^ tested (Figure 3A); however, we noted that there was less cleavage at −1 at lower concentrations of Mg^2+^ (Supplementary Figure S2). The Mg^2+^ requirement for optimal cleavage was found to be comparable with that for cleavage of pMini3bpUG (Figure 3B). However, compared with cleavage of precursor tRNAs and other longer model substrates [cf. Figures 5 and 6 in Ref. (14)], cleavage of the substrate lacking the nucleobase at −1 and the other pMini3bp variants (see later in the text) required a higher concentration of Mg^2+^ to reach optimal cleavage.

Next, we determined the kinetic rate constants at saturating Mg^2+^ concentration (800 mM). As shown in Table 1, deleting the nucleobase at −1 (pMini3bp*Abasic*G) resulted in a 4200-fold reduction in both k_obs_ and k_obs_/K^sto^ for cleavage at +1 compared with cleavage of pMini3bpUG at the same position. Given that pMini3bp*Abasic*G is cleaved at +1 and at −1, we compared the rate constants for cleavage at these positions. The k_obs_ values for cleavage at +1 and −1 were essentially the same, whereas k_obs_/K^sto^ was slightly higher for cleavage at −1. Following the argument that K^sto ^≈ K_d_ (see earlier in text), we calculated the K_d_ values using the data for cleavage at +1. This revealed that there was no significant effect on binding comparing pMini3bp*Abasic*G and pMini3bpUG.

**Table 1.** The kinetic constants k_obs_ and k_obs_/K^sto^ for cleavage of the various substrates by wild type *Eco* RPR

Substrate/[Mg^2+^]	k_obs_ (min^−1^)	k_obs_/K^sto ^(min^−1 ^µM^−1^)	K_d_ (µM)
pMini3bpUG
160 mM	0.86 ± 0.12	0.31 ± 0.12	2.8
800 mM	4.2 ± 0.48	1.6 ± 0.5	2.6
pMini3bp*Abasic*G			
800 mM
+1	0.0010 ± 0.000006	0.00038 ± 0.000053	2.6
−1	0.0012 ± 0.00004	0.0010 ± 0.00014	1.2
pMini3bpCG			
160 mM	0.0065 ± 0.0005	0.0006 ± 0.00012	11
800 mM	0.6 ± 0.3	0.19 ± 0.12	3.2
pMini3bpUA			
160 mM	0.8 ± 0.06	0.11 ± 0.01	7.3
800 mM	4.8 ± 0.95	3.7 ± 0.4	1.3
pMini3bpCA			
160 mM	0.29 ± 0.033	0.095 ± 0.015	3.1
800 mM	1.6 ± 0.2	1.3 ± 0.2	1.2
pMini3bp*Iso*CG			
160 mM	0.0046 ± 0.0003	0.0013 ± 0.0003	3.5
800 mM	0.062 ± 0.014	0.009 ± 0.001	6.9
pMini3bpAG			
800 mM	0.11 ± 0.061	0.034 ± 0.01	3.2
pMini3bpGG			
800 mM	0.0088 ± 0.0036	0.0012 ± 0.0002	7.3
pMini3bpInoG			
800 mM	0.22 ± 0.056	0.099 ± 0.026	2.2
pMini3bp2APG			
800 mM	0.016 ± 0.007	0.0088 ± 0.003	1.8
pMini3bpDAPG			
800 mM	0.025 ± 0.0048	0.013 ± 0.002	1.9
pMini3bpPuG			
800 mM	0.029 ± 0.0094	0.023 ± 0.007	1.3

The experiments were performed under single-turnover conditions at different Mg^2+^ concentrations at pH 6.1 as described in ‘Materials and Methods’. The final concentration of substrate was >10 nM. The concentration of *Eco* RPR was varied between 0.8 and 29 µM dependent on the substrate. For details regarding the calculation of K_d,_ see the main text. The data represent mean and experimental errors calculated from at least three independent experiments.

ND = not determined.

Taken together, these data show that the nucleobase at −1 is not essential for cleavage of the model substrate used here, but it does affect both the kinetics and accuracy of *Eco* RPR-mediated cleavage.

### The identity of residue −1 contributes to catalysis

We previously reported that the kinetic constant k_obs_ (see above Scheme 1) decreased 100-fold when U was replaced with C at the −1 position in the pMini3bp model substrate. These earlier experiments were done at 160 mM Mg^2+^, a non-optimal concentration [Table 1; cf. cleavage of pMini3bpUG and pMini3bpCG Figures 5 and 6 (14)]. Hence, to investigate the impact of the identity of the residue at −1, we now determined the optimal Mg^2+^ concentration for cleavage of these two substrates. A comparison of the Mg^2+^ profiles for cleavage of pMini3bpUG (Figure 3B) and pMini3bpCG (Figure 3C) revealed that cleavage of the latter required a higher Mg^2+^ concentration for optimal cleavage. This is consistent with our previous findings where we used pre-tRNA and longer model substrates [cf. Figures 5 and 6 in (14)].

Next, we determined k_obs_ and k_obs_/K^sto^ for cleavage of pMini3bpUG and pMini3bpCG at 800 mM Mg^2+^, a concentration that resulted in optimal cleavage for both substrates (see earlier in text). As shown in Table 1, we detected an almost 10-fold reduction in both these constants due to substitution of U with C at −1. However, it was not possible to determine whether the reduced cleavage of the C_−1_ variant was due to the identity of residue −1 or due to pairing between C_−1_ and G_+73_ (Figure 1B). Hence, we generated two new variants, pMini3bpUA and pMini3bpCA, where pairing between residues −1 and +73 was expected to be weaker and absent, respectively. This would allow us to study if k_obs_ and k_obs_/K^sto^ did differ for substrates with U or C at the −1 position.

An analysis of the cleavage as a function of Mg^2+^ concentration suggested that pMini3bpUA was cleaved more efficiently at position +1 (compared with pMini3bpCA) with no detectable cleavage at −1 (Figure 3B). The determination of k_obs_ and k_obs_/K^sto^ at two Mg^2+^ concentrations (160 and 800 mM; Table 1) revealed a 3-fold higher k_obs_ value for pMini3bpUA irrespective of Mg^2+^ concentrations. At saturating Mg^2+^ (800 mM), k_obs_/K^sto^ was also increased by a factor of three for pMini3bpUA suggesting that the K_d_ is not changed relative to pMini3bpCA. Rather the difference comparing U and C at −1 is related to a change in k_obs_ (Scheme 1).

In conclusion, the identity of residue −1 in *Eco* RPR-mediated cleavage contributes to catalysis with model hairpin loop substrate (see also later in text). U carries oxygen at position 4 (O4), whereas C has an exocyclic amine (N4) at this position (Figure 1B). Thus, the O4 of U at −1 likely confers only a minor advantage in RPR-mediated catalysis. This is consistent with our previous finding that the kinetic rate constants for cleavage of other model hairpin substrates with U_−1_/A_+73_ and C_−1_/A_+73_ under multiple turnover conditions are similar (13). Moreover, the replacement of U with C at −1 (pMini3bpUA and pMini3bpCA) resulted in a 3-fold effect on the kinetic constants, whereas almost a 10-fold effect was observed comparing cleavage of pMini3bpUG and pMini3bpCG. This indicates that the possibility to form a strong −1/+73 pair affects the kinetics of cleavage.

### The oxygen at C2 of U_−1_ and C_−1_ contributes to catalysis

Both U and C carry oxygen at C2 (O2) on the nucleobase (Figure 1C). To understand the influence of O2 on cleavage, we generated a pMini3bp derivative pMini3bp*Iso*CG in which O2 of U (or C) at position −1 is substituted with an exocyclic amine, 2NH_2_. *Eco* RPR_wt_ cleaved pMini3bp*Iso*CG preferentially (≈97%; Figure 2D) at the canonical site +1 irrespective of Mg^2+^ concentration but at a reduced rate relative to cleavage of pMini3bpUG. Moreover, as observed for the other pMini3bp variants, cleavage of pMini3bp*Iso*CG required high Mg^2+^ concentration for optimal cleavage (Figure 3A).

Determination of the kinetic constants at 800 mM Mg^2+^ revealed an ≈70- and >150-fold reduction in k_obs_ and k_obs_/K^sto^, respectively, compared with when pMini3bpUG was used. Using these data, K_d_ was calculated to be just ≈3-fold higher relative to the K_d_ value for pMini3bpUG (Table 1). These data suggest that O2 of U (or C) at −1 contributes significantly to *Eco* RPR-mediated catalysis of a model substrate and can, therefore, be considered to be a positive determinant.

### Presence of the exocyclic amine (2NH_2_) of G at −1 has a negative impact on cleavage at the canonical site +1

Bacteria also have pre-tRNAs with purines at −1; *E. coli* carry ≈13% and 8% A and G at −1, respectively (13). Processing studies with *Eco* RPR_wt_ (with and without the C5 protein) and yeast pre-tRNA^Ser^ derivatives show that cleavage between pyrimidines and purines is preferred to cleavage between two purines (31). Therefore, we decided to study the influence of a purine at −1 on substrate cleavage (all substrates have G at the +1 position; Figure 1). We were particularly interested to understand the impact of the exocyclic amine at C2 on the nucleobase (2NH_2_; Figure 1C) in RPR-mediated cleavage. We predicted that the 2NH_2_ at −1 (when present) would have a negative impact on cleavage by affecting the kinetic rate constants. Hence, six pMini3bp variants with different purines at −1 were generated (see Figure 1B) and subjected to cleavage by *Eco* RPR_wt_. As with the substrates discussed earlier in the text, we studied cleavage site recognition and Mg^2+^ dependence and determined the kinetic constants k_obs_ and k_obs_/K^sto^ at 800 mM Mg^2+^.

As seen with the other pMini3bp variants, cleavage of the substrates with the different purine derivatives at −1 required high Mg^2+^ concentration for optimal cleavage (Figure 3A, C and D). Moreover, introduction of G at −1 resulted in substantial cleavage at the alternative site −1 (Figure 2E and F). However, the frequency of cleavage at −1 depended on the Mg^2+^ concentration; there was less cleavage at −1 at lower Mg^2+^ concentrations (Supplementary Figure S2). When A was present at −1, cleavage occurred preferentially at the correct site +1 (Figure 2E and G). The substrate carrying inosine at −1 was also cleaved mainly at +1 (≈2% cleavage at −1; Figure 2G). As pMini3bpAG, this substrate lacks the 2NH_2_ group (compared with G at −1; pMini3bpGG versus pMini3bpInoG and pMini3bpAG; see Figure 1C). In contrast, the pMini3bp2APG and pMini3bpDAPG (with 2-AminoPurine and 2;6-DiAminoPurine at −1, respectively, Figure 1B and C) substrates were both cleaved more frequently at the alternative site −1 (Figure 2F; For pMini3bp2APG and pMini3bpDAPG there was no variation in cleavage site selection as a function of Mg^2+^ with the possible exception in the cleavage of pMini3bp2APG at high Mg^2+^, Supplementary Figure S2). As in the substrate with G at −1, both pMini3bp2APG and pMini3bpDAPG carry the 2NH_2_ group on the nucleobase. Moreover, a slight increase in the frequency of cleavage at −1 was detected when comparing cleavage of pMini3bpAG and pMini3bpPuG (Figure 2G). Cumulatively these data indicate that deletion of 6NH_2_ at −1 in a model substrate context marginally affect cleavage site recognition, whereas an exocylic amine (2NH_2_) at C2 on the purine base has a negative impact on the selection of the cleavage site.

Determination of the kinetic constants, k_obs_ and k_obs_/K^sto^ (Table 1) revealed that both were lower for substrates with A or G at −1 relative to those with U or C (cf. pMini3bpAG or pMini3bpGG versus pMini3bpUG or pMini3bpCG). This is particularly apparent when G is present at −1 (pMini3bpGG). In this case, there is an almost 500 - and >1000-fold reduction (for cleavage at +1) in k_obs_ and k_obs_/K^sto^, respectively, compared with cleavage of pMini3bpUG. For the substrate with A at −1 (pMini3bpAG versus pMini3bpUG), k_obs_ was reduced ≈40-fold and k_obs_/K^sto^ ≈50-fold. When the 2NH_2_ of G_−1_ (pMini3bpInoG versus pMini3bpGG) was deleted both k_obs_ and k_obs_/K^sto^ increased. By contrast, introduction of an exocyclic amine at C2 (pMini3bp2APG and pMini3bpDAPG) resulted in a decrease compared with cleavage of the substrate with A at −1 (pMini3bpAG; Table 1). Deletion of 6NH_2_ from the A at −1 (Figure 1C) decreased mainly the k_obs_ (3 - to 4-fold), whereas k_obs_/K^sto^ changed only marginally (cf. cleavage of pMini3bpAG and pMini3bpPuG; Table 1). A comparison of K_d_ values revealed only a modest increase (2 - to 3-fold) for the substrate with G at −1 relative to the substrates with U or C at −1. These data emphasize that the presence of 2NH_2_ on the purine base at −1 has a negative impact on the kinetics of cleavage, in agreement with our prediction. The presence of the exocyclic amine at C6 (6NH_2_) of A at −1, however, appears to have only a modest influence on cleavage efficiency.

In conclusion, RPR-mediated cleavage between purines in a model substrate context is unfavourable. An exocyclic amine at C2 on the purine base has a negative impact on cleavage both with respect to accuracy and kinetics of cleavage at +1. From our data, it also appears that a chemical group at C6 contributes to catalysis.

### Influence of the protein subunit of *Eco* RNase P

The RNase P protein, C5, binds to the 5′ leader of the substrate, and it affects binding and affinity of catalytic important Mg^2+^ (32–37). Also comparing cleavage with and without C5 of yeast pre-tRNA^Ser^ with G at −1 indicates that the presence of the C5 protein affects cleavage site selection (31). Therefore, the C5 protein is a positive factor for cleavage and selection of the cleavage site. Hence, we inquired whether addition of C5 to the reaction also influenced the choice of cleavage site when cleaving the model hairpin loop substrate with G (or A) at −1. Figure 2E shows that the reconstituted *Eco* RNase P holoenzyme cleaved pMini3bpGG at +1 with no apparent cleavage at −1. No change in choice of cleavage site was observed for cleavage of the substrate having A at −1 with RPR or with the reconstituted *Eco* RNase P holoenzyme. However, the substrate with A at −1 was cleaved more efficiently compared with cleavage of the substrate with G at −1 also in the presence of the C5 protein (cf. lanes 5 and 6 Figure 2E).

These data show that the C5 protein suppressed cleavage at the alternative site −1 also in the case of model substrates carrying G at −1. This further indicates that C5 has a strong positive effect on the cleavage site recognition process.

### Interaction between residue −1 and 248 in the RPR

**Figure 4.**
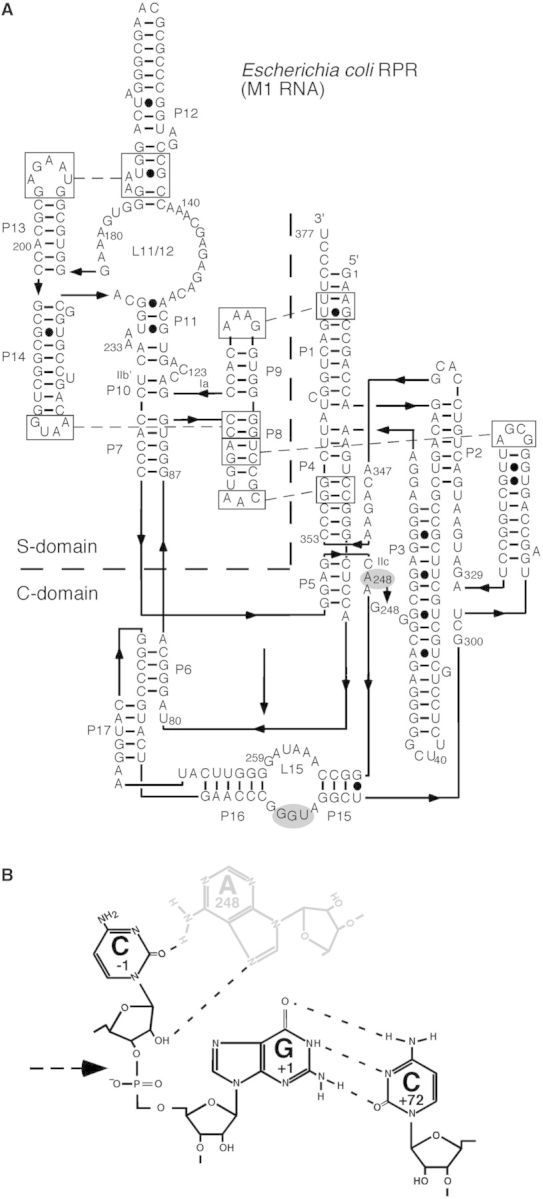
Secondary structure model of *Eco* RPR and a model of the N_−1_/A_248_ interaction. (**A**) Secondary structure of *Eco* RPR_wt_ according to Massire *et al.* (39). Residue A_248_ that was changed to G is indicated in grey. (**B**) A putative model of the N_−1_/A_248_ interaction. Black and grey residues mark substrate and RPR residues, respectively, and the dashed arrow marks the scissile bond.

**Figure 5.**
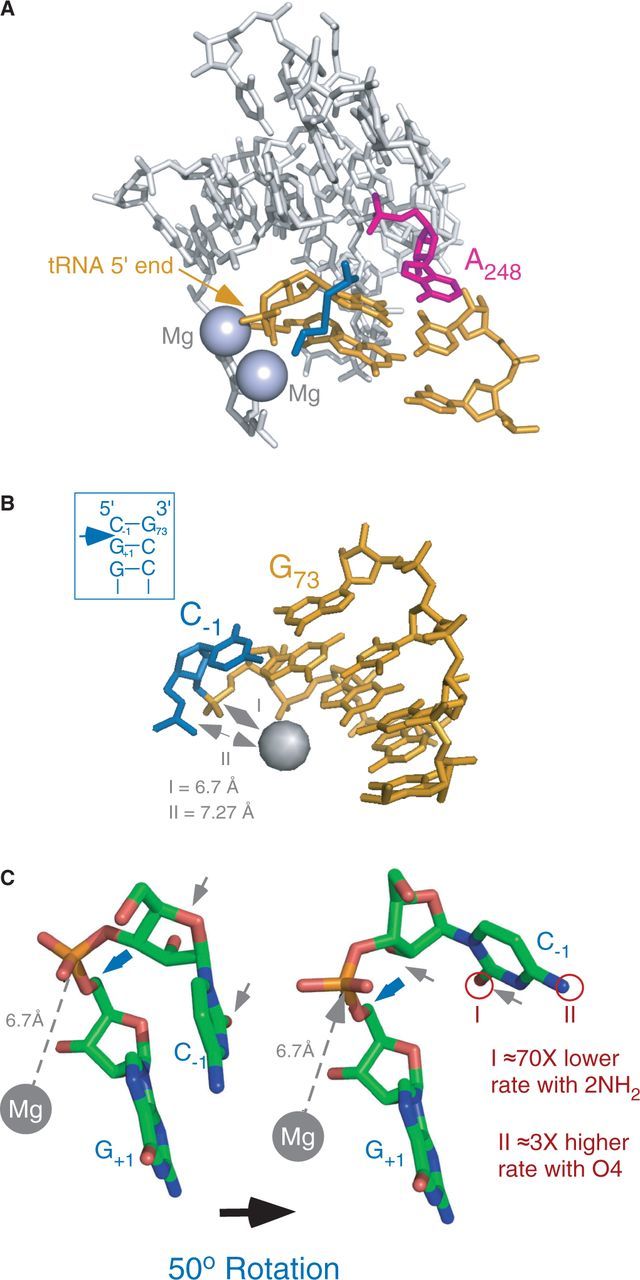
The structure of the RPR active site and model of the RNase P cleavage site. (**A**) The active site of RPR from the PDB structure 3OKB (11). Residues G_+1_ of the 5′ matured tRNA and A_248_ (*E. coli* numbering) are marked as indicated. The grey spheres corresponds to the Mg^2+^ ions observed in the structure, and the blue structure represents the 5′ leader that was soaked into the crystal. (**B**) Model of the RNase P cleavage site with C_−1_/G_+73_ and G_+1_/C_+72_. The model is part of the SRP RNA structure [PDB code 1LNT; (40)]. The phosphorous atom to be attacked is marked with the grey dashed double arrow. The light blue sphere represents Mg^2+^ bound in the minor groove and is positioned at a distance of 6.7 Å relative to phosphorous atom I, and 7.27 Å relative to phosphorous atom II; I and II are referred to as P(+1) and P(−1) in the main text. This distance allows for a nucleophilic attack on P(+1) by an activated H_2_O coordinated to a divalent metal ion (41,42). The boxed hairpin structure shows the sequence of SRP RNA that mimics the RNase P cleavage site. The blue arrow marks the cleavage site. (**C**) Model of the RNase P cleavage site showing the conformational change in the substrate that facilitates the nucleophilic attack on P(+1). The model was prepared using the structure shown in panel B and rotating the P-O5′ phosphoester bond (marked with a blue arrow) 50°. The rotation displaces the pro-Sp non-bridging oxygen and exposes the P(+1)-atom to an inline hypothetical nuclephilic attack by a Mg^2+^-activated H_2_O (grey dashed arrow). The grey arrows mark the 2′OH and O2 of C_−1,_ and I and II refer to the changes in cleavage rates as a result of changing O2 to 2NH_2_ and O4 to 4NH_2_, respectively.

The A at position 248 in the RPR (Figures 1A and 4) has been suggested to interact with the −1 residue in the substrate (9). In the crystal structure of RNase P in complex with tRNA, which represents a post cleavage state, A_248_ is positioned near the 5′ termini of the tRNA [(11); Figure 5A]. Nucleotide analogue-modification interference studies suggest that the Hoogsteen surface of A_248_ plays an important role in the interaction with the substrate (38). As shown here, substitution of O2 of U (or C) at −1 with NH_2_ affects the kinetics of cleavage (see earlier in text; pMini3bp*Iso*CG). Previous data reveal that the 2'OH of residue −1 contribute to catalysis [(7) and references therein]. The O2 and 2'OH of residue −1 are exposed on the same surface (Figure 5B; see later in text). Together this opens for the possibility that N7 and 6NH_2_ of A_248_ are hydrogen-bonded to the 2'OH and O2 of residue −1, respectively, in the transition state (Figure 4B). If this hypothesis is valid, changing of A_248_ to G in the RPR would restore the interaction between *Iso*C at −1 and 248 by forming hydrogen bonding between 2NH_2_ of *Iso*C and O6 of the G at 248. As a consequence, the rate of cleavage of pMini3bp*Iso*CG should increase using an RPR variant with G at 248 compared with cleavage with RPR_wt_. To test this prediction, we generated *Eco* RPR_G248_. Structural probing revealed no significant overall structural difference compared with *Eco* RPR_wt_ [Supplementary Figure S3; see also (19). We note the appearance of additional cleavages, located between well-established lead(II)-induced sites IV and V, when *Eco* RPR_G248_ was subjected to RNase T1 cleavage. This might indicate a slight change in the structure in this region].

As shown in Figure 2C and D, *Eco* RPR_G248_ cleaved pMini3bpUG and pMini3bp*Iso*CG preferentially at the correct site, +1. For pMini3bp*Iso*CG, we also observed ≈9% cleavage at −1, which was higher at lower Mg^2+^ (Supplementary Figure S2). Moreover, compared with *Eco* RPR_wt_, the G_248_ variant showed similar Mg^2+^ profiles in cleavage of pMini3bpUG and pMini3bp*Iso*CG (Figure 3E). Determinations of the apparent rate constants (k_app_) at 800 mM Mg^2+^ revealed that *Eco* RPR_G248_ cleaved these two substrates with slightly reduced rates relative to the wild type (Table 2). Importantly, the rate of cleavage did not improve as predicted. Rather, the rate was lower with *Eco* RPR_G248_ compared with *Eco* RPR_wt_. These findings, therefore, suggest that 6NH_2_ of A_248_ is not engaged in hydrogen bonding with O2 of U (or C) at −1 or at least not in the context of a model substrate (see also the ‘Discussion’).

**Table 2.** Apparent rates, k_app_, for cleavage of pMini3bpUG and pMini3bp*Iso*CG with *Eco* RPR_wt_ and *Eco* RPR_G248_

Substrate	*Eco* RPR variant	
	Wt	G_248_
pMini3bpUG		
+1	1.5 ± 0.03	0.98 ± 0.06
pMini3bp*Iso*CG		
+1	0.026 ± 0.002	0.011 ± 0.0004
−1	ND	0.001 ± 0.00007

k_app_ = pmol cleaved per min. The experiments were performed in buffer C containing 800 mM Mg^2+^ at 37°C. The concentrations of RPRs and substrates were 4 and 0.02 µM, respectively. The data represent mean and experimental errors calculated from at least three independent experiments.

ND = not determined.

## DISCUSSION

Here we provide data that the nucleobase, the identity and specific chemical groups of the nucleobase at position −1 in a model substrate contribute significantly to catalysis in the *E. coli* RPR alone reaction. In particular, our data suggest that the carbonyl oxygen (O2) at C2 on C and U at −1 is a positive factor, whereas a purine (A or G) at −1 and the presence of an exocyclic amine at C2 (2NH_2_) have a negative impact on the kinetics and accuracy of cleavage. The nucleobase at −1 is, however, not essential for cleavage although it affects the kinetic rate constants, k_obs_ and k_obs_/K^sto^. It contributes to catalysis by stabilizing the transition state, at least in the context of a model substrate, and this corresponds to ≈5.1 kcal [using k_obs_ values for cleavage of pMini3bp*Abasic*G and pMini3bpUG at +1 and ΔΔG = −RTlnk_obs_(Δ_−1_)/k_obs_(U_−1_); (43)]. Of these 5.1 kcal, the contribution of the O2 (with U at −1) amounts to 2.6 kcal [ΔΔG = −RTlnk_obs_(*Iso*C_−1_)/k_obs_(U_−1_)], which corresponds roughly to one hydrogen bond. In accordance with this is that the presence of 2NH_2_ on a purine at −1 destabilizes the transition state with up to 2 kcal (using k_obs_ values for cleavage of pMini3bpInoG and pMini3bpGG at +1). Interestingly, Baidya *et al.* (44) used modified pyrimidines at position 17 (5′ residue of the cleavage site) in the hammerhead ribozyme and showed that an abasic residue at position 17 almost abolished cleavage. They also suggested that O2 of C17 makes an important contribution to catalysis by stabilizing the transition-state structure. The recently solved structure of the *Schistosoma mansoni* hammerhead ribozyme suggests hydrogen bonding between O2 of C17 at the cleavage site and 6NH_2_ of A13 that presumably helps to stabilize the transition state (45). However, the recent crystal structure of bacterial RNase P in complex with tRNA (11) does not reveal any structural information about the nature of the interaction between residue −1 in the substrate and the RPR (see later in text). We conclude that the O2 (in the case of U or C) at position −1 in a model substrate acts as a positive determinant in *Eco* RPR-mediated catalysis whereas 2NH_2_ (in the cases of U, C or purines) at this position has a negative impact.

### Comparing cleavage of pre-tRNA versus model hairpin loop substrates

Studies where pre-tRNA^Asp^ was used (9,46) show modest changes in both k_obs_ and K_d_ (except for the variant with C at −1 where K_d_ increased 230-fold, possibly owing to the C at −1 being paired with the G at +73 in the substrate). In contrast, cleavage of model substrates (this report) caused an almost 500-fold change in k_obs_ (for G at −1) with only small changes in K_d_. Moreover, model substrates with G at −1 (or purines with 2NH_2_ or where the nucleobase had been deleted at −1) resulted in significant cleavage at −1 whereas the other −1 variants were cleaved mainly at +1 (Figure 2 and Supplementary Figure S2). This is in contrast to the U at −1 substitution in pre-tRNA^Asp^, which did not change the cleavage site. However, cleavage of pre-tRNA^Asp^ at the alternative site −1 was observed when residue −1 was changed together with replacement of the 2'OH with 2'H at −1, i.e. two cleavage site determinants had been changed (9,46). A comparison of the structures of pre-tRNA^Asp^ and pMini3bpUG reveals striking similarities at and near their respective cleavage sites

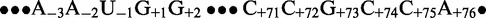

[black circles correspond to residues that are not shown and the canonical cleavage site is between U_−1_ and G_+1_; Figure 1 and (46)]. The T-loop/stem, known to affect site selection (14,22), is absent in the pMini3bp model substrates. This makes them dependent on the remaining determinants, e.g. the −1 residue (Figure 1A). Together this argues that the impact of the −1 residue in a pre-tRNA (all ribo) context is obscured by the presence of several residues and regions that interact with the RPR on formation of the RPR-substrate complex. Nevertheless, for both model and pre-tRNA substrates, the base at −1 as well as its identity influences cleavage efficiency and site recognition but it is not essential for cleavage. However, as shown in this report using model substrates, specific chemical groups on the −1 nucleobase influence both transition-state stabilization and accuracy in *Eco* RPR-mediated cleavage.

We have reported elsewhere that the presence of an exocyclic amine (2NH_2_) on the nucleobase at +1 plays an important role for cleavage at the correct (+1) site (17). Our present data show that replacement of U (or C) at −1 with purines with 2NH_2_ in a model substrate had a negative impact on the kinetic constants with significant cleavage at the alternative site −1. On the basis of these data we suggest that 2NH_2_ (in the case of G) at −1 functions as a positive determinant for cleavage at −1, i.e. at the site immediately 5′ of a purine carrying 2NH_2_ (see Figure 1). At the same time, it acts as a negative factor for cleavage at +1. This would explain why the efficiency of cleavage between two G residues is low (31). Most bacterial tRNA^His^ precursors [and pre-tRNA^SeCys^; Ref (47)] carry G at both −1 and +1, and this model also provides one reason to why bacterial RPR cleaves pre-tRNA^His^ and pre-tRNA^SeCys^ at −1 and not between two G-residues [reviewed in (6)]. However, we have to consider that addition of the C5 protein, which interacts with the substrate 5′ leader (see earlier in text), resulted in cleavage mainly at +1 even with G at −1 in the model substrate (Figure 2E). It is conceivable that this is due to G_−1_ in the model substrate is not being paired with a C at +73, in contrast to pre-tRNA^His^ and pre-tRNA^SeCys^ in which the G at −1 is paired to a C. The presence of a G_−1_/C_+73_ pair in different precursor substrates plays an important role for cleavage at −1 (6). Also, *E. coli* pre-tRNA(valU) and pre-tRNA(thrW) carry G at −1 (48) but in those two cases G_−1_ is unlikely to pair with the discriminator base (which is A in both cases) at position +73. Another factor to consider is that the difference in cleavage site selection of the G_−1_ model substrate with and without the C5 protein may be an effect of the higher Mg^2+^ concentration used in the ‘RPR alone reaction’ (Supplementary Figure S2). Evidently, the role of the C5 protein in the cleavage site recognition process in the processing of different precursors requires further studies. The present study together with our previous data (17), however, indicates the importance of the presence of G (and the 2NH_2_) 3′ of the scissile bond at least in the ‘*Eco* RPR alone reaction’. In this context, we note the importance of the exocyclic amine of a guanosine marking the cleavage site in the reaction catalyzed by the group I ribozyme (49), indicating similarities with group I RNA and RPR-mediated cleavage.

### Interaction between the −1 residue in the substrate and *Eco* RPR and positioning of Mg^2+^ at the cleavage site

A_248_ has been suggested to play a key role in the interaction with residue −1; this interaction is referred to as the N_−1_/A_248_ interaction [(9,38,46,50); see also (6) and Figure 1A]. The crystal structure of the RNase P-tRNA complex, which represents the post-cleavage state, does not provide any information about the interaction between the RPR and the −1 residue (11). Although it cannot be excluded that the Watson–Crick surface of A_248_ is involved in pairing with residue −1 as suggested elsewhere (9,46), we consider this unlikely. The reasons are: (i) as mentioned earlier in the text, the identity of residue −1 varies and must be taken into account; (ii) N3-methyl-U at −1 in a model substrate did not affect the kinetic rate constants, k_obs_ and k_obs_/K^sto^, to any significant extent compared with a substrate with an unmodified U at −1 (51); and (iii) a model substrate with C_−1_/G_+73_ is cleaved with increased frequency at −1 (and not a decrease in cleavage at the alternative site −1, as would be predicted if it was base pairing between −1 and 248) when A_248_ in *Eco* RPR_wt_ was substituted with a G (19). Alternatively, given that the Hoogsteen surface of A_248_ is facing the tRNA 5′-end in the crystal structure (Figure 5A) it is possible that N7 and 6NH_2_ interact with specific groups of the −1 residue. This would be consistent with nucleotide analogue-modification interference studies, which suggest that the Hoogsteen surface of A_248_ plays an important role on interaction with the substrate (38). Here a possibility is that 6NH_2_ and N7 of A_248_ interact with O2 (in the case of U and C) and the 2'OH (Figure 4B; see also below) of residue −1, respectively. The lack of rescue in cleavage of pMini3bp*Iso*CG with RPR_G248_ argues against this alternative; however, we cannot conclusively exclude this possibility (see later in the text).

RPR catalysis depends on divalent metal ions, preferentially Mg^2+^, and two Mg^2+^ are positioned close to the tRNA 5′-end in the RNase P-tRNA complex (Figure 5A). In our model, the Mg^2+^ that activates the nucleophilic water is positioned 6.7 Å from the phosphorous between −1 and +1 [P(+1) in Figure 5B] in the substrate (7,52,53). The distance between this Mg^2+^ and the phosphorous between −1 and −2 [P(−1) in Figure 5B] is just 0.57 Å longer. Hence, changing the identity of residues or chemical groups in the vicinity of the cleavage site can affect the charge distribution. This would then shift the positioning of Mg^2+^ that influences the rate of cleavage and site selection. This model is consistent with our data where substitutions of the nucleobase and chemical groups at −1 or at position 248 in the RPR affected the rate constants and site selection [see also (19); it also raises the possibility that the change in charge distribution owing to the presence of G at 248 is why we did not observe rescue of cleavage of pMini3bp*Iso*CG with *Eco* RPR_G248_]. In addition, deletion of the exocyclic amine (2NH_2_) of the G at +1 in a model substrate influenced the charge distribution at the cleavage site (17). Substitutions in the T-loop also influenced both the rate and site of cleavage in metal(II)-ion induced hydrolysis of yeast tRNA^Phe^ (54,55).

### A model for displacement of the −1 residue in the *Eco* RPR-substrate complex

For bacterial RPR, the 2'OH at −1 plays an important role for catalysis (6–8). It contributes 2.3 kcal to the stabilization of the transition state in cleavage of a hairpin loop model substrate using the same formula discussed earlier in text and data from Brännvall and Kirsebom (51). As reported here, the contribution of the carbonyl oxygen O2 of C (or U) at −1 in a model substrate to transition-state stabilization was 2.6 kcal (see earlier in text). The 2'OH and O2 of C at −1 in a model of the cleavage site were exposed on the same surface (marked with grey arrows in Figure 5C). We cannot exclude that one or both these groups form hydrogen bonds with the RPR in the transition state. However, available data suggest that the 2'OH at −1 acts as an outer (or inner) sphere ligand for Mg^2+^ [(24,56–59); but see also (46) for an alternative interpretation]. On the basis of this, it is therefore conceivable that also the carbonyl oxygen of C (or U) at −1 is involved in Mg^2+^ binding although the crystal structure does not confirm this. Nevertheless, on RPR-substrate complex formation, where residue +73 pairs with U294 in the RPR, the C_−1_/G_+73_ (when present) opens. This conformational change is likely accompanied with displacement of the C at −1 and positioning of A_248_ such that it stacks over the G_+1_/C_+72_ base pair (Figure 5A). We propose that displacement of the −1 residue is the result of a 50-degree rotation (Figure 5C) such that O2 (in the case of U or C at −1) and the 2'OH are facing away from the scissile phosphate. As a consequence, the Mg^2^^+^-activated H_2_O bound to the substrate in the vicinity of the phosphorus [P(+1)] to be attacked is positioned for an in-line attack [Figure 5; Ref (7)]. At the same time, the 2'OH at −1 is prevented from attacking the phosphorus, which would lead to cleavage products with faulty ends, i.e. a 5'OH and a 2′; 3′ cyclic phosphate (24). This model is consistent with a conformational-assisted mechanism of cleavage but needs to be confirmed by a structure of RNase P in complex with its precursor substrate.

## SUPPLEMENTARY DATA

Supplementary Data are available at NAR Online.

## FUNDING

Funding for open access charge: Swedish Research Council; Uppsala RNA Research Center (Swedish Research Council Linneus support).

*Conflict of interest statement*. Leif A. Kirsebom is on the board of directors of Bioimics AB.

## Supplementary Material

Supplementary Data
