# Supplementary material for: Transition-state stabilization in Escherichia coli ribonuclease P RNA-mediated cleavage of model substrates
Source: Nucleic Acids Res. 2013 Oct 3;42(1):631–42. doi: 10.1093/nar/gkt853 (PMC3874170; doi:10.1093/nar/gkt853)
Supplement: Supplementary Data [file supp_42_1_631__index.html]

Transition-state stabilization in Escherichia coli ribonuclease P RNA-mediated cleavage of model substrates — Transition-state stabilization in Escherichia coli ribonuclease P RNA-mediated cleavage of model substrates — Supplementary Data 

# Transition-state stabilization in *Escherichia coli* ribonuclease P RNA-mediated cleavage of model substrates

## Supplementary Data

files

**Files in this Data Supplement:**

- Supplementary Data - pdf file
