# Supplementary material for: Transition-state stabilization in Escherichia coli ribonuclease P RNA-mediated cleavage of model substrates
Source: Nucleic Acids Res. 2013 Oct 3;42(1):631–42. doi: 10.1093/nar/gkt853 (PMC3874170; doi:10.1093/nar/gkt853)
Supplement: Supplementary Data [file supp_gkt853_nar-01130-h-2013-File007.pdf]

Supplementary information

Chase experiment

The cleavage reaction was performed in 20 μl at 37 °C in buffer C (50 mM MES, pH 6.1, 0.8 M NH_4OAc) supplemented with 800 mM $Mg(OAc)_2$. The concentration of *Eco* RPR was 3.2 μM while the substrate concentration was 0.02 μM. The *Eco* RPR and substrate were preincubated as outlined in Materials and Methods and 10 sec after initiation of the reaction 10 μl of the reaction mix was transferred to 2 ml of dilution buffer (50 mM MES, pH 6.1 at 37°C, 0.8 M NH_4OAc and 800 mM $Mg(OAc)_2$). At each time point, 200 μl aliquots were removed and mixed with 700 μl of 99.5% EtOH, then 20 μl of 3 M NaOAc, pH 5.1 and 2 μl of glycogen (stock concentration 20 mg/ml). As a control an identical experiment without chase was run in parallel and terminated at indicated times. The samples were stored at -20 °C overnight. The ethanol precipitated samples were collected by centrifugation for 40 min at 16000g in a microcentrifuge (Eppendorf) at 4°C. Each sample was dried and dissolved in 10 μl of water and 20 μl of stop solution (8.4 M Urea, 1.2 mM EDTA, 0.036% bromophenol blue, 0.036% xylene cynal) and subjected to gel-electrophoresis on 22% denaturing polyacylamide gels (10 mM Tris-borate, pH 7.5, 1 mM EDTA and 7 M Urea) to separate the cleavage products. The products were visualized using a PhosphorImager (Molecular Dynamics 400) and quantification was done using the ImageQuant software. For further details, see Stage-Zimmermann and Uhlenbeck (28), Sinapah *et al.* (24), and Chen *et al.* (29) in the reference list in the main text).

Structural probing of Eco *RPR_{wt} and* Eco *RPR_{G248}*

Lead(II)-induced cleavage and limited RNase T1 digestion under native conditions were conducted with *Eco* RPR_{wt} and *Eco* RPR_{G248} that had been 3'-end labeled with [^{32}P]pCp essentially as described elsewhere (see below, Refs 1-3). Briefly, approximately 2 pmols of

labeled RPR in 10 μl was pre-incubated for 10 min at 37°C in 50 mM Tris-HCl (pH 7.5), 100 mM NH_4Cl and 10 mM $MgCl_2$. The mixture also contained 4 μM tRNA (no label). Cleavage was initiated by the addition of freshly prepared $Pb(OAc)_2$ to a final concentration of 2.5 mM and the reaction was stopped after 10 min. In the case of digestion with RNase T1, the RPR was pre-incubated as described above. One unit RNase T1 was added and the reaction mixture was incubated on ice for 10 min. The reactions were stopped after 10 min and the products were analyzed on and 8% denaturing polyacrylamide gel.

Supplementary References

1. Kufel,J. and Kirsebom,L.A. (1996) Residues in Escherichia coli RNase P RNA important for cleavage site selection and divalent metal ion binding. *J. Mol. Biol.,* **263**, 685-698.

2. Kufel,J. and Kirsebom,L.A. (1998) The P15-loop of *Escherichia coli* RNase P RNA is an autonomous divalent metal ion binding domain. *RNA*, **4**, 777-788.

3. Brännvall,M., Mikkelsen,N.E. and Kirsebom,L.A. (2001) Monitoring the structure of Escherichia RNase P RNA in the presence of various divalent metal ions. *Nucleic Acids Res*., **29**, 1426-1432.

Supplementary figure legends

Figure S1

Result from a dilution experiment to assess if $k_{-1} >> k_{obs}$ during *Eco* RPR-mediated cleavage of pMini3bpUG

Under single-turn over conditions, the absence of rate increase of cleavage post-dilution suggests that the dissociation rate constant $k_{-1} >> k_{obs}$ (the rate constant of cleavage, Scheme 1 in the text). Hence, $K^{sto} \approx K_d$ and K_d corresponds to the binding constant. The experiment was performed as described in Supplementary information. The filled squares represent the control experiment without chase while the filled circles represent the result after dilution (chase).

Figure S2

Fraction of cleavage at -1 of selected pMini3bp variants with modified residues at -1 by *Eco* RPR_{wt} and *Eco* RPR_{G248} at different divalent Mg^{2+} concentrations.

The reactions were performed at 37°C in buffer C containing indicated concentrations of Mg^{2+}. The concentration of *Eco* RPR_{wt} and *Eco* RPR_{G248}, and substrate were as described in Materials and Methods.

The data represent mean and experimental errors calculated from at least three independent experiment.

Figure S3

Structural probing of *Eco* RPR_{wt} and Eco_{G248}

A. Lead(II)-induced hydrolysis and RNase T1 digestion of *Eco* RPR_{wt} and Eco_{G248}. The Roman numerals mark the lead(II)-induced cleavage sites (see also Supplementary Ref 3). The reactions were conducted as outlined in Supplementary information. Lanes 1 and 4, incubation of RPRs without the addition of Pb^{2+} or RNase T1; lanes 2 and 5, incubation of RPRs in the presence of $Pb(OAc)_2$ and lanes 3 and 6, partial digestion of the RPRs with RNase T1. The asterisk marks a band that appears when *Eco* RPR_{G248} was subjected to digestion with RNase T1.

B. Secondary structure of *Eco* RPR_{wt} according to Massire *et al.* (Ref 54 in the main text). The black arrowheads mark the lead(II)-induced cleavage sites (Ia to V). Residue A_{248} that was changed to G is indicated in grey.

Figure S1

Illustration of a typical result from a dilution experiment to assess if k_{-1}>>k_2 (see Scheme 1 in the main text) during *Eco* RPR_{wt}-mediated cleavage of pMini3bpUG.

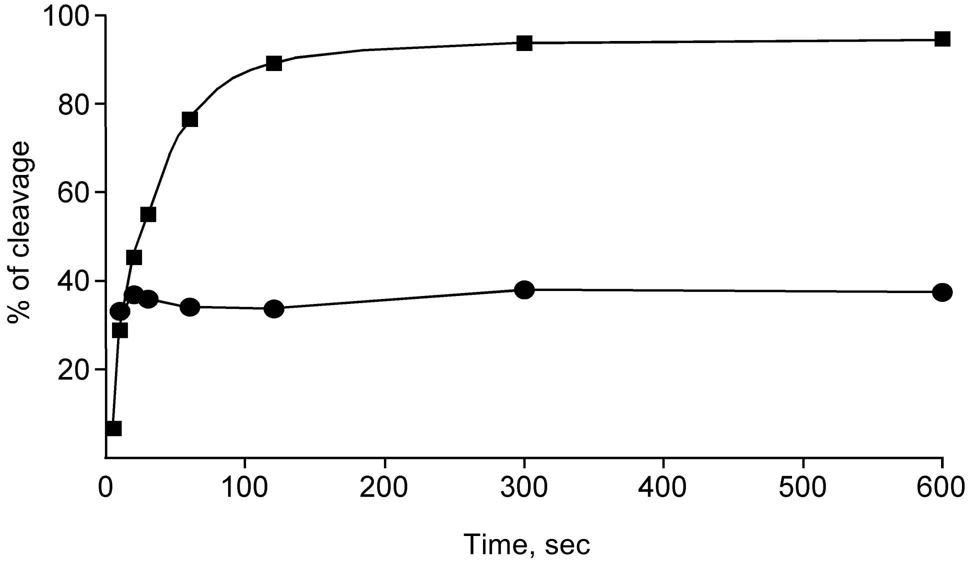

Figure S2

A. Fraction of cleavage of pMini3bp*Abasic*G at -1 with *Eco* RPR_{wt} at different Mg^{2+}.

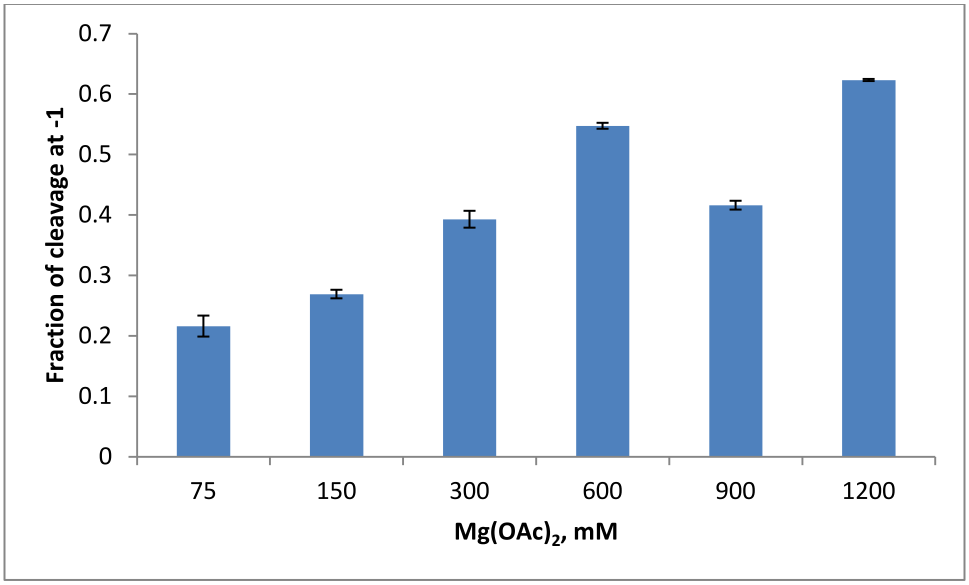

B. Fraction of cleavage of pMini3bpGG at -1 with *Eco* RPR_{wt} at different Mg^{2+}.

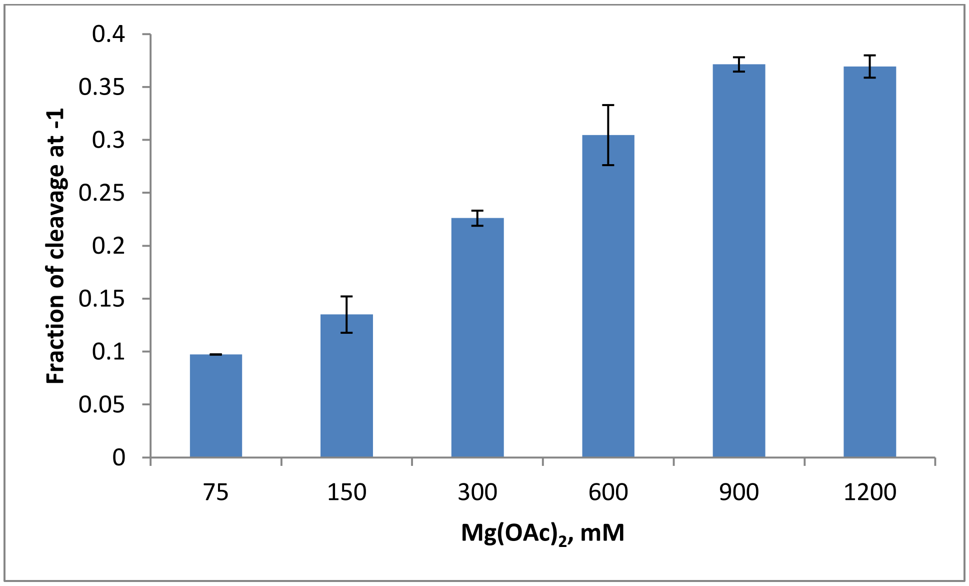

C. Fraction of cleavage of pMini3bp2APG at -1 with *Eco* RPR_{wt} at different Mg^{2+}.

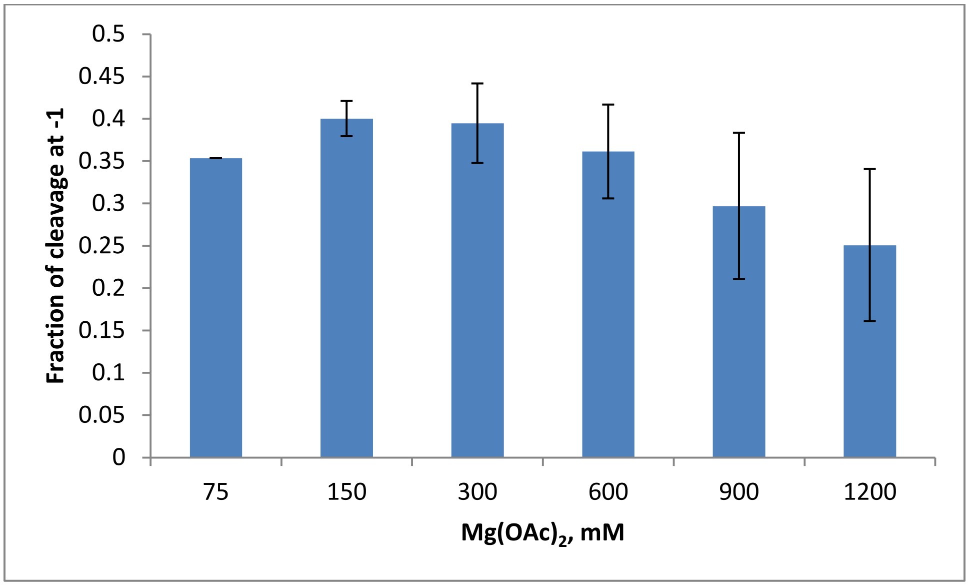

D. Fraction of cleavage of pMini3bpDAPG at -1 with *Eco* RPR_{wt} at different Mg^{2+}.

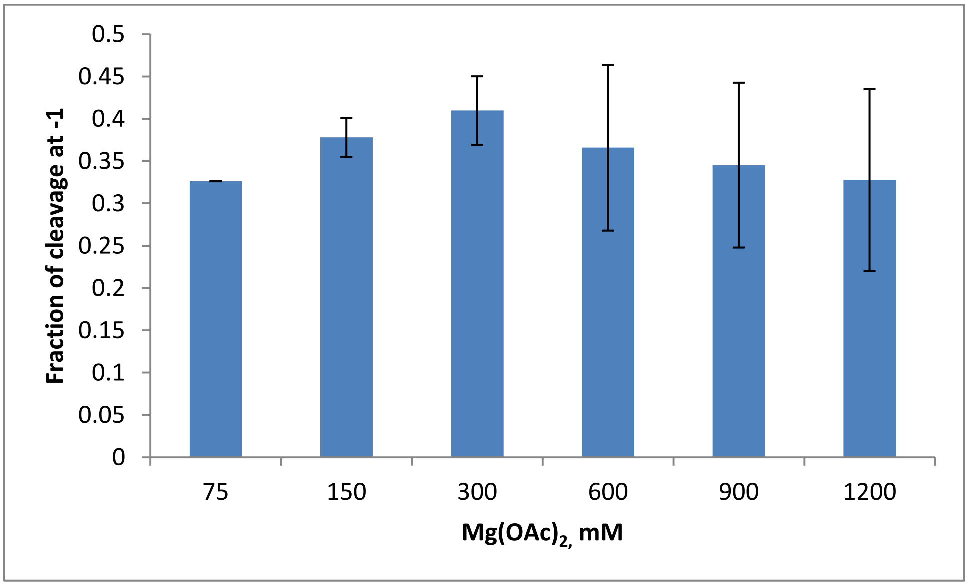

E. Fraction of cleavage of pMini3bp*Iso*CG at -1 with *Eco* RPR_{G248} at different Mg^{2+}.

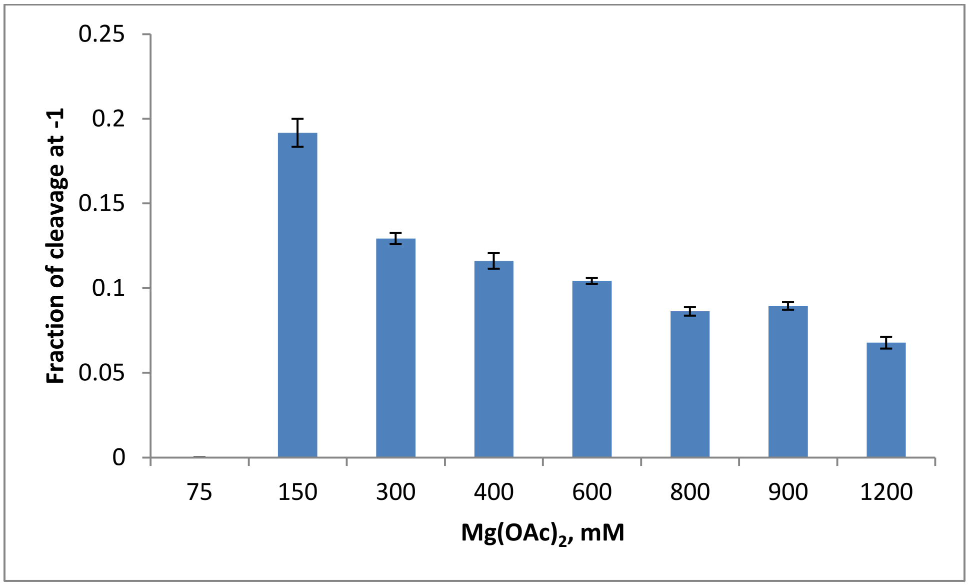

Figure S3

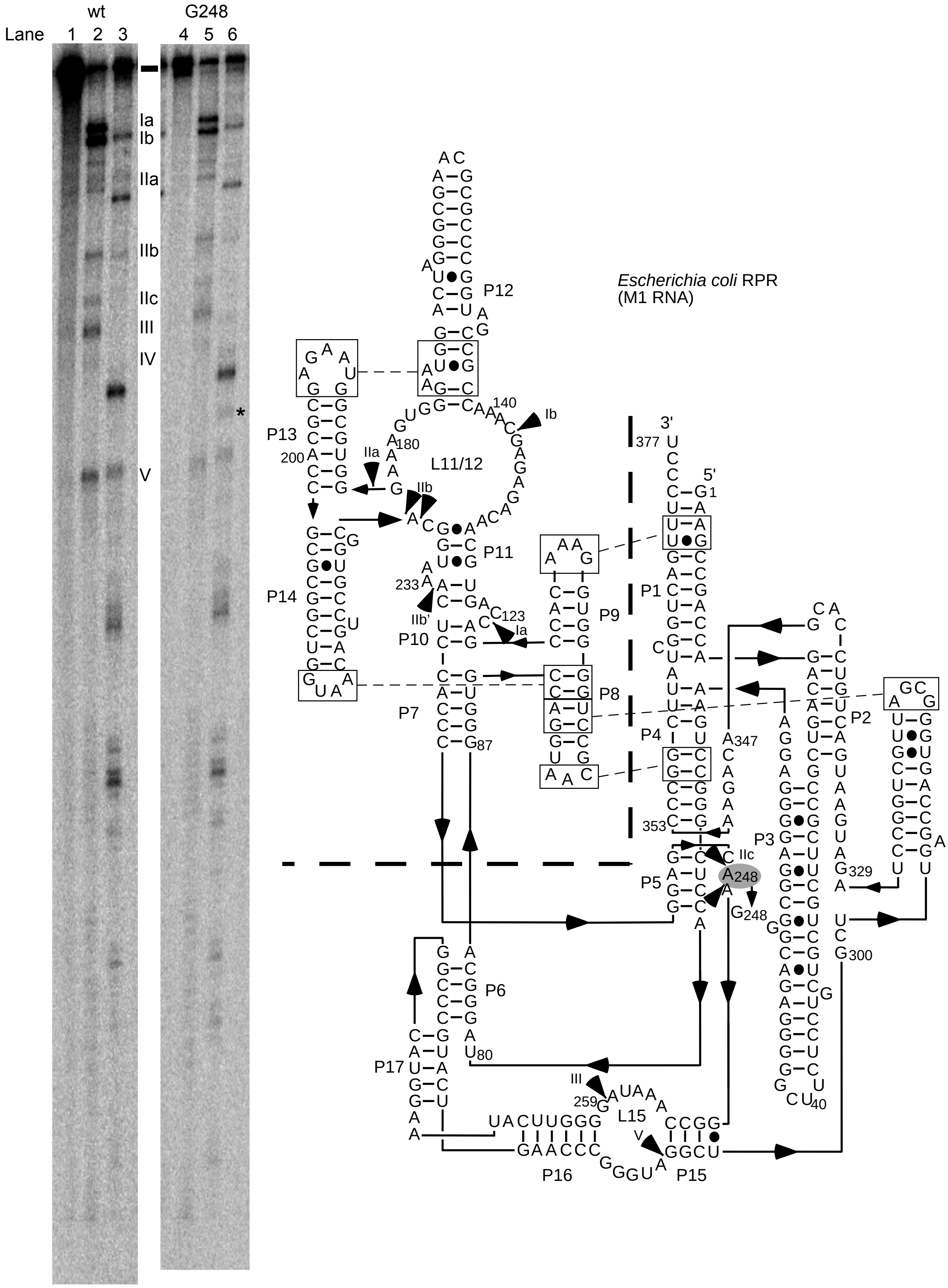
wt
G248
Lane 1 2 3 4 5 6
Ia
Ib
IIa
IIb
IIc
III
IV
V
*
Escherichia coli RPR
(M1 RNA)
P1
P2
P3
P4
P5
P6
P7
P8
P9
P10
P11
P12
P13
P14
P15
P16
P17
L11/12
L15
IIb'
3'
5'
A248
G248
